# Distribution of Acid Sensing Ion Channels in Axonal Growth Cones and Presynaptic Membrane of Cultured Hippocampal Neurons

**DOI:** 10.3389/fncel.2020.00205

**Published:** 2020-07-07

**Authors:** Xiaoyan Liu, Can Liu, Jiamin Ye, Shuzhuo Zhang, Kai Wang, Ruibin Su

**Affiliations:** ^1^State Key Laboratory of Toxicology and Medical Countermeasures, Beijing Key Laboratory of Neuropsychopharmacology, Beijing Institute of Pharmacology and Toxicology, Beijing, China; ^2^School of Pharmacy, North China University of Science and Technology, Tangshan, China; ^3^State Key Laboratory of Proteomics, National Center of Biomedical Analysis, Beijing, China

**Keywords:** acid-sensing ion channels, growth cones, hippocampal neurons, presynaptic membrane, axon, synapse, neurotransmitter release

## Abstract

Although acid-sensing ion channels (ASICs) are widely expressed in the central nervous system, their distribution and roles in axonal growth cones remain unclear. In this study, we examined ASIC localization and function in the axonal growth cones of cultured immature hippocampal neurons. Our immunocytochemical data showed that native and overexpressed ASIC1a and ASIC2a are both localized in growth cones of cultured young hippocampal neurons. Calcium imaging and electrophysiological assay results were utilized to validate their function. The calcium imaging test results indicated that the ASICs (primarily ASIC1a) present in growth cones mediate calcium influx despite the addition of voltage-gated Ca^2+^ channels antagonists and the depletion of intracellular calcium stores. The electrophysiological tests results suggested that a rapid decrease in extracellular pH at the growth cones of voltage-clamped neurons elicits inward currents that were blocked by bath application of the ASIC antagonist amiloride, showing that the ASICs expressed at growth cones are functional. The subsequent immuno-colocalization test results demonstrated that ASIC1a and ASIC2a are both colocalized with Neurofilament-H and Bassoon in mature hippocampal neurons. This finding demonstrated that after reaching maturity, ASIC1a and ASIC2a are both distributed in axons and the presynaptic membrane. Our data reveal the distribution of functional ASICs in growth cones of immature hippocampal neurons and the presence of ASICs in the axons and presynaptic membrane of mature hippocampal neurons, indicating a possible role for ASICs in axonal guidance, synapse formation and neurotransmitter release.

## Introduction

Acid-sensing ion channels (ASICs), members of the amiloride-sensitive degenerin/epithelial Na^+^ channel superfamily, are largely expressed in the mammalian nervous system (Waldmann et al., 1997). Six different ASIC subunits (1a, 1b, 2a, 2b, 3, and 4), encoded by four genes, have been cloned (Kellenberger and Schild, 2015). These subunits form homomeric and heteromeric channels vary in expression within tissues and organs, and activation of pH values and function under physiological and pathological conditions (Lingueglia et al., 1997; Wemmie et al., 2003; Grunder and Pusch, 2015). Under normal physiological states, ASICs are involved in learning and memory, anxiety and fear responses (Wemmie et al., 2002, 2004; Zha et al., 2006; Du et al., 2014; Kreple et al., 2014). Under pathological states, they have also been shown to be involved in ischemic stroke (Xiong et al., 2004, 2006; Chu and Xiong, 2012), epileptic seizures (Ziemann et al., 2008), pain perception (Wemmie et al., 2013) and many other neuropsychiatric diseases.

The ASIC1a and ASIC2a subunits are widely expressed in neurons of central nervous system, exhibiting particularly high abundance in the cerebral cortex, amygdala, olfactory bulb, cerebellum and hippocampus (Price et al., 2014). Owing to the dependence of ASIC2a homomers on a low pH midpoint (∼4.5) for activation, ASIC1a homomers and ASIC1a/ASIC2a heteromers are major functional effectors that determine the magnitude of acid-induced responses. Among them, only ASIC1a homomer activation mediates the acid-induced intracellular Ca^2+^ concentration increase that is closely related to physiological and pathological functions. At the subcellular level, ASIC1a and ASIC2a are both enriched in the soma, dendrites and dendritic spines, the sites of excitatory neurotransmission in the brain (Wemmie et al., 2002, 2003; Alvarez de la Rosa et al., 2003).Structurally, ASIC1a, as postsynaptic membrane proton receptor, affects the density, length and maturity of dendritic spines (Alvarez de la Rosa et al., 2003; Zha et al., 2006; Yu et al., 2018). Functionally, ASIC1a plays important roles in the induction of excitatory postsynaptic currents and miniature excitatory postsynaptic currents (mEPSCs) and thus influences long-term potentiation (LTP) in the hippocampus and amygdala (Du et al., 2014; Chiang et al., 2015) and the induction of long-term depression (LTD) in the insular cortex and hippocampus (Li et al., 2016; Mango and Nistico, 2019). Behaviorally, mice without ASIC1a show impairments in multiple forms of learning, such as cerebellum-dependent eyeblink conditioning (Wemmie et al., 2002), amygdala and stria terminalis-dependent fear learning and memory (Du et al., 2014; Ziemann et al., 2009; Chiang et al., 2015), extinction learning of conditioned taste aversion (Li et al., 2016) and striatum-dependent procedural motor learning. The subcellular distribution of ASIC2a is essentially consistent with that of ASIC1a. Like ASIC1a, ASIC2a is also localized at dendritic spines (Zha et al., 2009). In addition, ASIC2 facilitates ASIC1a localization and function in dendritic spines. The regulatory effect of ASIC2a on ASIC1a may occur by it facilitating the formation of the *N*-linked glycans in ASIC1a (Jiang et al., 2017). In addition, ASIC2a is associated with epilepsy susceptibility, ischemic stroke and light-induced retinal degeneration (Ettaiche et al., 2004; Jiang et al., 2017; Zhang et al., 2017).

However, whether ASIC1a and ASIC2a are distributed in the growth cones of immature neurons during development is not known. In addition, whether ASIC1a and ASIC2a are presynaptically localized in mature neurons is controversial (Hruska-Hageman et al., 2002; Alvarez de la Rosa et al., 2003; Zha et al., 2006). In this study, we demonstrated that the ASIC1a and ASIC2a subunits are present in axonal growth cones from 3 days *in vitro* (DIV3) to DIV6 hippocampal neurons. Moreover, we demonstrate that these ASICs are functional in the growth cones and are also expressed in the axons and presynaptic active zones of mature (DIV14) neurons, suggesting that they may regulate neurotransmitter release.

## Materials and Methods

### Preparation of Hippocampal Neurons and Adeno-Associated Virus Transduction

Primary neuronal cultures were prepared as previous report (Ruscher et al., 2002). Simply speaking, hippocampal explants isolated from E18 rats of either sex were digested with 0.25% trypsin for 30 min at 37°C, followed by trituration with pipette in plating medium (DMEM with 10% fetal bovine serum plus 10% horse serum). Dissociated neurons were plated onto 24-well plates or 6-well plates (Corning, United States) coated with poly-D-lysine at a density of 1 × 10^5^ cells or 2 × 10^6^ cells per well according to different experimental requirements. After culturing for 3 h, media were changed to neurobasal medium (Gibco, Grand Island, NY, United States) supplemented with 2% B27. Growth cones were examined only from DIV3 to DIV6. Because if beyond this age range, *in vitro* isolated neurons will form synaptic connections that leads to growth cones difficult to be recognized even in low density cultures. According to selection criteria of Wang et al. (2011), only cultured neurons that had clear axonal growth cones located down-stream of the spraying flow, relative to the cell body, were chosen for electrophysiology and calcium imaging experiments. cDNAs of *ASIC1* (GenBank: NM_024154.2) and *ASIC2* (GenBank: NM_001034014.1) were synthesized and cloned into adeno-associated virus (AAV)-dj type backbone (Brainvta, Wuhan, China). The experimental results from hippocampal neuronal cultures were obtained from at least three independent cultures per group (*n* ≥ 10). In experiments involving overexpression of ASIC1a and ASIC2a *in vitro*, DIV0 or DIV6 cultures were transduced with AAV-dj-ASIC1-mCherry and AAV-dj-ASIC2-eGFP (viral titers:1 × 10^13^ vg/ml, 0.5 μl/ml culture media). After transduction, DIV6 or DIV14 cultures were used for growth cone and synapse immunofluorescence experiments, respectively.

### Immunofluorescence

Neurons grown on coverslip were washed with PBS three times. They were fixed with 4% paraformaldehyde for 20 min, which were subsequently washed with PBS (3 × 5 min). Thereafter, they were permeabilized and blocked with 0.3% Triton X- 100 plus 3% BSA for 40 min at room temperature. And then, neurons were incubated with primary antibodies overnight at 4°C and washed with PBS (3 × 5 min). Alexa Fluor 488 or 592 secondary antibodies were put on for 1 h at room temperature followed by repeated washing in PBS (3 × 5 min). Finally, the cells were mounted on slides, and the stained sections were observed with Olympus fluorescent microscope (Olympus BX51, Tokyo, Japan) and confocal fluorescence microscopy (Ultraview vox, PerkinElmer, United States). Primary antibodies included Guinea pig anti-ASIC1 antibody (1:100, alomone labs), rabbit anti-ASIC2 antibody (1:100, Millipore), rhodamine phalloidin (1:250, Invitrogen), mouse Neurofilament-H antibody (1:50, Cell Signaling Technology), mouse Bassoon antibody (1:100, Novus Biologicals), rabbit GFP antibody (1:100, Cell Signaling Technology) and rabbit mCherry antibody (1:100, Cell Signaling Technology). Secondary antibodies contained goat anti-mouse, anti-rabbit or anti-guinea pig conjugated secondary antibody (1:200, Invitrogen). Supplementary Material showed the specificity of ASICs antibodies (Supplementary Figure 3).

### Protein Preparations and Western Blot

Neurons were collected and lysed in RIPA lysis buffer. Protein concentration was determined by Bio-Rad Protein Assay (Pierce^TM^ BCA Protein Assay Kit; Thermo Fisher Scientific, Rockford, IL, United States). Lysates (10 μg) were resolved by denaturing 8% sodium dodecyl sulfate-polyacrylamide gel electrophoresis and transferred to polyvinylidene difluoride membranes and then blocked with 5% (w/v) skim milk in PBS for 1 h at room temperature. The blocked membranes were subsequently probed with rabbit anti-ASIC2 antibody (1:200, Millipore) or mouse anti-ASIC1 antibody (1:500, Millipore) or β- actin (1:1000; Invitrogen) antibodies at 4°C overnight. After the membrane had been washed three times with TBST [20 mmol/L Tris–HCl (pH 7.4), 0.15 mol/L NaCl, 0.1% Tween 20], it was incubated for 1 h at room temperature with IRDye 800CW goat anti-rabbit or anti-mouse secondary antibody (1:5000). After the membrane had been washed with TBST, bands of protein on the membrane were visualized using the enhanced chemiluminescence detection (Li-Con, Odyssey, Hong Kong, China).

### Patch-Clamp Recording

The whole-cell patch clamp recordings were performed at room temperature; currents were measured with an Axopatch-200B (Molecular Device, Sunnyvale, CA, United States) and recorded with pClamp 8.2 software (Molecular Device). The output was digitized with a Digidata 1440 converter (Molecular Device). Patch pipettes were made by a horizontal puller (Model P-97, Sutter Instrument) from borosilicate glass and had resistances between 5 and 10 MΩ after perfusion of internal solution through the pipette. Neurons in the glass coverslip dishes were placed in a recording chamber and visualized with the phase contrast inverted fluorescence microscope (BDS400, Optec, China). The image acquisition system is DV630 (Optec, China). Experiments were performed at a holding potential of −70 mV. After gigaohm seal formation, the membrane was disrupted. Data were low-pass-filtered at 2 kHz, sampled at 10 kHz, and acquired with the gap-free protocol. The liquid junction potential between internal and external solutions was −5 mV on average and was used to correct for the recorded membrane potential. The pipette solution was composed of the following (in mmol/L): KCl 140, NaCl 5, MgCl_2_ 2, EGTA 5, K_2_ATP 2, HEPES 10 (pH 7.4), and the bath solution contained (mmol/L): NaCl 150, KCl 5, MgCl_2_ 2, CaCl_2_ 2, glucose 10, HEPES 10 (pH 7.4). 20 μmol/L CNQX, 100 μmol/L DL-APV, and 10 μmol/L bicuculline were added in order to inhibit glutamate- and GABA-induced currents. 4-Morpholinoethanesulfonic acid (MES) was used instead of HEPES to buffer bath solution pH 5.0. Tetrodotoxin (TTX) (1 μmol/L) was added to the solution to block voltage sensitive sodium channels and spontaneous action potentials. Application of pH 5.0 extracellular solution was performed using a Picospritzer III (Parker Hannifen) pressure application system. Pressure and lasting time were 4–6 psi and 1 s, respectively. Experiments were carried out at room temperature (20–24°C).

### Calcium Imaging

Primary cultured hippocampal neurons of the rat were used 3–6 days after plating. After loading with Fluo-4 AM (Invitrogen), coverslips were placed in a chamber at room temperature with a HBSS solution containing (mmol/L): NaCl 137.9, KCl 5.3, CaCl_2_ 1.26, Na_2_HPO_4_ 0.34, MgSO_4_ 0.4, glucose 5.6, and KH_2_PO_4_ 0.44 and NaHCO_3_ 4.17. TTX 1 μmol/L was added and the pH adjusted to 7.35–7.40 with 10 mol/L NaOH. pH 5.0 external solution was puffed to axonal growth cones using a Picospritzer III pressure application system (4–6 psi for 1 s). Pressure and lasting time guaranteed locally accurate administration to growth cones that prevented diffusion of pH 5.0 external solution toward the cell body. Calcium transients were imaged using a laser scanning confocal microscopy (Ultraview vox, PerkinElmer, United States) in conjunction with EMCCD camera (C9100, PerkinElmer, United States) and Volocity 6.1.1software (PerkinElmer, United States). Images were taken at 200 ms frame intervals. The exposure at the 10 s time point in Figure 4 began at the onset of pH 5.0 external solution application. Calcium transients were quantified using Excel and Volocity software. Regions of interest (ROIs) were chosen to encircle the entire growth cone structure, and background ROIs were selected close proximity to the growth cone. △F/F measurements were performed using the following formula: (F–F_min_)/F_max_, where F was the mean pixel fluorescent value in circumscribed growth cones, and F_min_ was the average of darkest pixel fluorescent value. F_max_ was the average of brightest pixel fluorescent value. All images were background (baseline levels) subtracted. The first five mean background pixel intensity were the baseline levels.

### Chemicals and Data Analysis

The following pharmacological compounds were used, as described in text. In control electrophysiology and calcium imaging experiments, NMDARs, AMPARs and GABARs were blocked by adding 100 μmol/L DL-APV, 20 μmol/L CNQX, and 10 μmol/L bicuculline to the extracellular solution and introduced into the recording chamber by perfusion. Voltage-sensitive calcium channels (VSCCs) were blocked with nimodipine, MVIIA and ω-agatoxin IVA, which block L-type, N-type, and P/Q-type voltage-gated calcium ion channel (VGCC) blockers, respectively. Replenishment of intracellular calcium stores was blocked using 10 μmol/L cyclopiazonic acid (CPA). Psalmotoxin 1 (PcTX1) was used to block ASIC1a homomeric channels. All reagents were dissolved in distilled water, except for nimodipine and CPA, which were dissolved in dimethylsulfoxide (DMSO). All of above reagents are from Tocris.

The data were analyzed by Clampfit 10.0 (Molecular Device, United States) and Origin 8.0 (Microcal Software, United States) software programs. All data were presented as mean ± SEM. Statistical significance was assessed using Prism5.0 software. The Student’s *t*-test analysis was used to assess the differences between the means of two groups. One-way ANOVA followed by Tukey’s multiple comparisons test was performed to assess differences among more than two groups. *p*-value < 0.05 was considered to be significant.

## Results

### ASICs Localization at the Growth Cones of Immature Cultured Hippocampal Neurons

**FIGURE 1:**
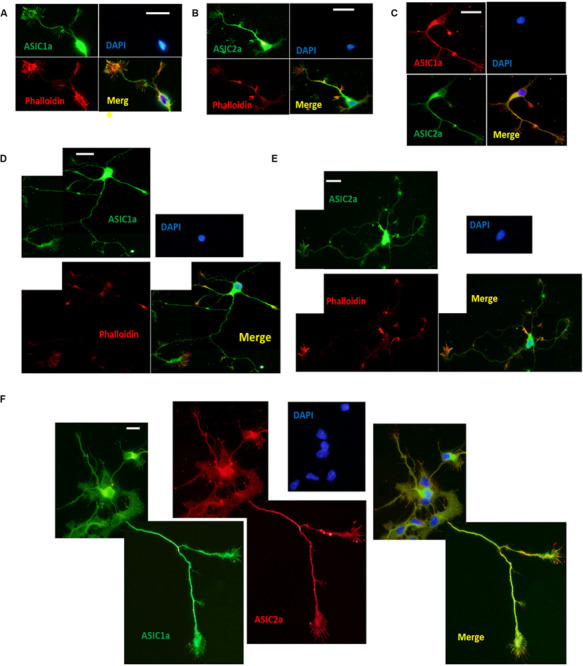
Endogenous ASIC1a and ASIC2a are expressed in growth cones of DIV3-DIV6 hippocampal neurons. **(A,B)** DIV3 primary hippocampal cultures were coimmunostained for ASIC1a and phalloidin, ASIC2a and phalloidin, respectively. ASIC1a and ASIC2a display the distribution throughout the cell and are present at axonal growth cones. **(C)** Distribution of ASIC1a and ASIC2a is almost the same at DIV3 primary hippocampal neurons. **(D,E)** DIV6 primary hippocampal cultures were coimmunostained for ASIC1a and phalloidin, ASIC2a and phalloidin, respectively. **(F)** Distribution of ASIC1a and ASIC2a is almost the same at DIV6 primary hippocampal neurons. Scale bars, 20 μm.

To investigate the localization of ASICs at growth cones, we performed immunocytochemistry for endogenous ASIC1a and ASIC2a, which are the primary ASIC subunits expressed in the central nervous systems in DIV3–DIV6 hippocampal neurons, using anti-ASIC1a and anti-ASIC2a antibodies. To facilitate the identification of growth cones, the neurons were coimmunostained with phalloidin (Huang et al., 2017). Immunocytochemistry was performed at DIV3 and DIV6 due to easy morphological recognition. ASIC1a and ASIC2a labeling revealed that at DIV3, both subunits were distributed throughout the soma, dendrites, axons, and growth cones (Figures 1A,B). ASIC1a and ASIC2a were present at both the peripheral (filopodia and lamellipodia) and central (microtubules) domains of growth cones. Immunofluorescence colocalization assay results showed that ASIC1a had almost the same distribution as ASIC2a at DIV3 (Figure 1C). Subsequently, we further assessed whether ASIC1a and ASIC2a remained localized in growth cones at DIV6, when growth cone construction is clear before neuron maturation. We observed that the results at DIV6 (Figures 1D,F) were the same as those at DIV3. These results indicate that ASIC1a and ASIC2a are both expressed in growth cone structures that appear to be in the process of forming early presynaptic contacts with dendrites.

The above results showed that ASIC1a and ASIC2a are endogenously expressed in growth cones. Subsequently, we assessed the effect of exogenous overexpression of these proteins. Constructs encoding mCherry fluorescent protein conjugated to rat ASIC1 and enhanced green fluorescent protein (eGFP) conjugated to rat ASIC2 were separately packaged into an adeno-associated virus (AAV)-dj type (cultures transduced with these AAV-djs will be referred to as AAV-dj-ASIC1a-mCherry and AAV-dj-ASIC2a-eGFP, respectively).

**FIGURE 2:**
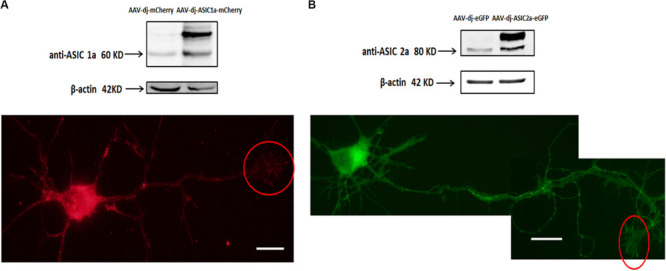
Transduced ASIC1a and ASIC2a is expressed at the axonal growth cones. **(A)** Primary hippocampal cultures were transduced with AAV-dj-mCherry and AAV-dj-ASIC1a-mCherry. The above panel shows that the protein expression (AAV-dj-ASIC1a-mCherry) of ASIC1a (60 kD) and fusion mCherry (28.8 kD) was higher than that of the proteins in the control groups (AAV-dj-mCherry). The below panel shows that ASIC1a overexpression signal was also distributed at the axonal growth cones (red circle). **(B)** Primary hippocampal cultures were transduced with AAV-dj-eGFP and AAV-dj-ASIC2a-eGFP. The above panel shows that the protein expression (AAV-dj-ASIC2a- eGFP) of ASIC2a (80 kD) and fusion eGFP (27 kD) was higher than that of the proteins in the control groups (AAV-dj-eGFP). The below panel shows that exogenous overexpression of ASIC2a was also localized at the axonal growth cones (red circle). Scale bars, 20 μm.

Cultures were transduced at DIV0 and imaged and immunoblotted at DIV6. The overexpression of ASIC1a and ASIC2a was quantified by western blot analysis. We observed that at the protein level, ASIC1a and ASIC2a expression was significantly increased in the AAV-dj-ASIC1a-mCherry and AAV-dj-ASIC2a-eGFP cultures compared with that observed in the AAV-dj-mCherry and AAV-dj-eGFP control cultures (Figure 2). The molecular weights of ASIC1a and ASIC2a were approximately 60 and 80 kD, respectively. In addition to an enhancement of the protein bands, the expression of the proteins fused with mCherry (28.8 kD) and eGFP (27 kD) was higher and more clearly observed than that of the proteins in the respective control groups. The ASIC1a and ASIC2a fluorescence signals were detected in growth cones at DIV6. In addition to the soma, dendrites and axons, ASIC1a and ASIC2a were also distributed at growth cones. These findings showed that the ASIC1a and ASIC2a proteins were also expressed in growth cones in the exogenous system.

### ASICs Are Functional at Axonal Growth Cones

**FIGURE 3:**
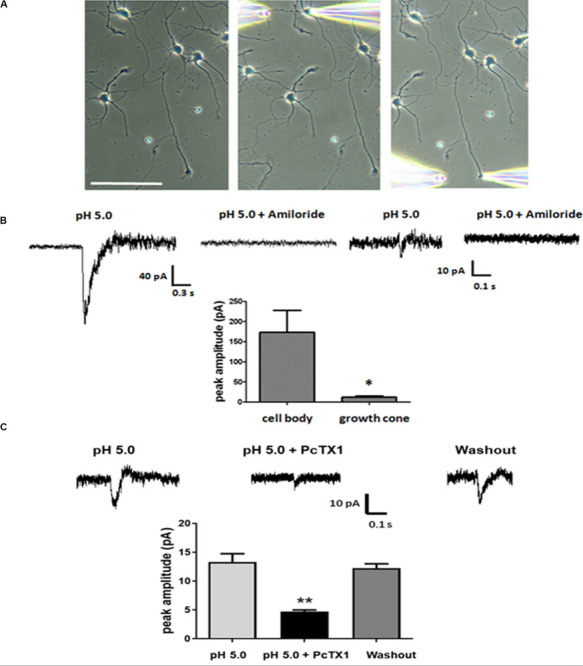
Functional ASICs are present at axonal growth cones. DIV5 and DIV6 primary hippocampal neurons were voltage-clamp at –70 mV. pH 5.0 acid extracellular solution was applied to the cell body and the growth cone by pressure application (4–6 psi, 1 s). **(A)** The image of a representative neuron from which the currents were recorded in response to acid extracellular solution administration. The positions of the application pipette and recording pipette are shown. Scale bar, 100 μm. **(B)** Electrophysiological recordings were performed in the presence of TTX (1 μmol/L). To test the specificity of the ASIC-mediated response at cell body and growth cones, 200 μmol/L Amiloride was added to the acid extracellular solution. Average peak amplitudes for ASICs currents at the cell body and growth cones were 132 ± 40.8 pA (*n* = 7) and 14.1 ± 1.6 pA (*n* = 11), respectively. Data are means ± SEM and analyzed by unpaired Student’s *t*-test, *p < 0.05. **(C)** Blockade of growth cone ASIC currents by 50 nmol/L PcTX1 venom (*n* = 4). Data are means ± SEM and analyzed by one-way ANOVA followed by Tukey’s multiple comparisons test, **p < 0.01.

Our experiments thus far established that ASICs are present at the growth cones of DIV3-DIV6 primary hippocampal neurons *in vitro*. Next, we wanted to assess whether the ASICs were functional. Low-density primary hippocampal neurons (1 × 10^4^/ml) were cultured on the slice to allow individual cells to be easily recognized and not influenced by other cells. The neurons at DIV5-DIV6 were selected by a whole-cell voltage-clamp technique at −70 mV holding potential. Then, an extracellular solution at a pH of 5.0 was locally applied to the axonal growth cones by pressure application (4–6 psi) for 1 s. To restrict the acidic extracellular solution application to the growth cone, only the growth cones that were positioned downstream and at least 100 μm away from the cell body were selected for analysis. The pipette resistances ranged from 6 to 8 MΩ. In addition, the pipettes were positioned 30–40 μm from the growth cones. In this drug delivery pattern, the acidic extracellular solution was directed specifically toward the growth cones, and the dendrites and cell body were not exposed to the acidic solution, although the distal axon was not excluded (Figure 3A). For each cell selected, the acidic extracellular solution was first applied onto the cell body as a control and then repositioned to the growth cone as illustrated in Figure 3A. Representative traces from a single cell, including the cell body and growth cone, are shown in Figure 3B. The administration of the ASIC antagonist amiloride (200 μmol/L) completely eliminated the inward currents induced by the application of the acidic solution to the cell body and growth cone. DL-APV, CNQX, and bicuculline were added to the extracellular solution to inhibit glutamate- and GABA-induced currents. In addition, these experiments were performed in the presence of 1 μmol/L TTX to prevent spontaneous excitatory postsynaptic currents. The results of this experiment showed that the observed response was mediated by ASICs. The average peak amplitude of the ASIC-like current of the growth cone was approximately 14.1 ± 1.6 pA (*n* = 11), whereas the current amplitude of the cell body was 132 ± 40.8 pA (*n* = 7). The above data suggest that ASICs are functional at axonal growth cones. PcTX1 is a tarantula venom peptide that inhibits ASIC1a homomeric channels (Escoubas et al., 2000; Xiong et al., 2004) but not ASIC2a/1a heteromeric channels or ASIC2a homomeric channels (Chen et al., 2006). PcTX1 venom (50 nmol/L) reversibly blocked the peak amplitude of the growth cone ASIC currents by ∼66% (Figure 3C) (*n* = 4), indicating significant contributions of homomeric ASIC1a to total acid-activated currents. However, other ASIC currents should also be included, such as ASIC2a/1a heteromeric currents and ASIC2a homomeric currents.

### ASICs Activation at Growth Cones Promotes Calcium Influx

**FIGURE 4:**
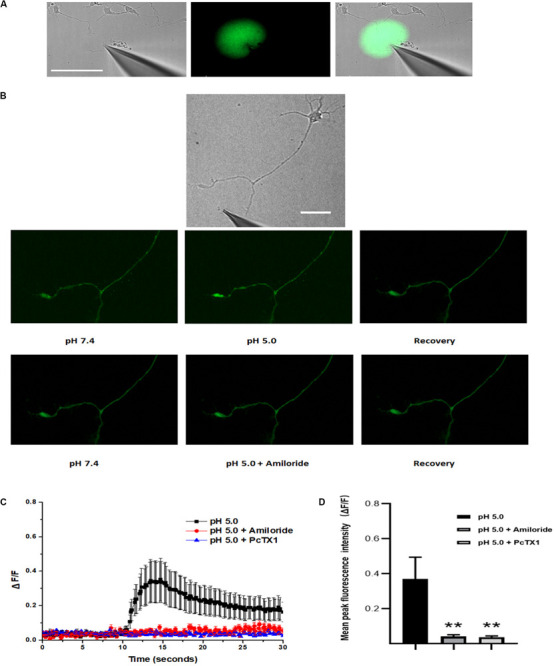
Activation of ASICs at axonal growth cones leads to calcium influx. DIV3-DIV6 hippocampal neurons were loaded with Fluo-4 AM calcium indicator. **(A)** DIC image of the experimental system (left). To examine the area of the pressure application, a spray delivery electrode was loaded with a mixture of Alexa Fluor 488 and HBSS (volume ratio 1:10) and applied to the growth cone. Fluorescent signals of the application were pseudo-colored based on pixel intensity (middle). The fluorescent image was digitally merged with a DIC image of the experimental system for visualization purposes (right). Scale bar, 100 μm. **(B)** Representative DIC image of the experimental system (top). Local application of acid solution to the growth cone induces calcium influx (middle). Adding 200 μmol/L amiloride to the extracellular solution in advance eliminates the ASICs-mediated calcium influx (bottom). Scale bar, 40 μm. **(C)** ΔF/F analysis reveals an significant increase in fluorescence (calcium transients) at the growth cone after local application of acid solution (black square) (*n* = 12). Application of 200 μmol/L (*n* = 8) amiloride and 50 nmol/L PcTX1(*n* = 4) to the extracellular solution blocked ASICs-mediated calcium transients (red circle and blue triangle), respectively. Experiments were performed in the presence of TTX, VSCCs blockers (nimodipine, MVIIA and ω-agatoxin IVA), and CPA (which blocks the replenishment of intracellular calcium stores). **(D)** Mean peak fluorescence intensity (ΔF/F) is statistics from experiment illustrated in **(C)**. Data are means ± SEM, and analyzed by one-way ANOVA followed by Tukey’s multiple comparisons test, **p < 0.01.

ASIC1a channel activation at growth cones promotes localized calcium influx. The ASIC1a and ASIC2a subunits are major ASIC subunits expressed in hippocampal neurons. Among the homomeric ASIC1a, homomeric ASIC2a and heteromeric ASIC1a/2a channels, only homomeric ASIC1a channels are calcium permeable. Therefore, to obtain additional evidence for the localization of functional homomeric ASIC1a channels in growth cones, we used confocal microscopy to image calcium transients in the growth cones of DIV3-DIV6 hippocampal neurons. The high-affinity cell-permeable fluorescent calcium indicator Fluo-4 AM was loaded into the neurons. A pH 5.0 external solution was applied to the growth cone and cell body by pressure application. To examine the spatial specificity of the local application system, a mixture of Alexa Fluor 488 and HBSS in a volume ratio of 1:10 in the spray delivery electrode was applied to the growth cone. The growth cone was specifically exposed with this method, which did not influence the cell body, dendrites and proximal axon (Figure 4A). Obvious dynamic calcium transients were visualized using confocal fluorescence microscopy at growth cones (Figure 4B). The application of 200 μmol/L amiloride and 50 nmol/L PcTX1 before the application of the acidic solution prevented these calcium transients (Figures 4B,C), verifying functional expression of homomeric ASIC1a channels. To eliminate potential contributions from VGCCs to the calcium transients, VGCCs antagonists, including nimodipine, MVIIA and ω-agatoxin IVA were added to the extracellular solution. In addition, TTX (1 μmol/L) was added to the solution to block voltage-gated sodium channels and thus inhibit action potentials. To block the replenishment of intracellular calcium stores, 20 μmol/L CPA was also added to the HBSS. In the presence of all of the above inhibitors, the application of the pH 5.0 acidic solution still caused significant local calcium transients at growth cones with an average peak increase in fluorescence intensity (ΔF/F, *n* = 12) of 37.1 ± 12.4%. In addition, the fluorescence intensity was decreased by treatment with 200 μmol/L amiloride and 50 nmol/L PcTX (Figures 4C,D). Calcium imaging results demonstrated that homomeric ASIC1a channels mediate the localized Ca^2+^ influx. These results also show that ASICs, or at least ASIC1a channels, are functional in growth cones.

### ASICs Localization Later in Mature Hippocampal Neurons

**FIGURE 5:**
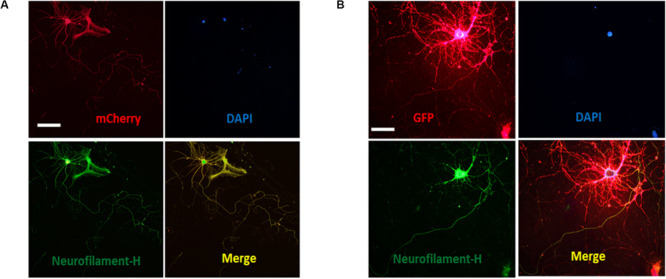
Distribution of ASICs on axons of cultured mature hippocampal neurons. **(A)** Co-staining of hippocampal neurons with anti-mCherry (ASIC1a, red) and Neurofilament-H (green) antibodies. Overlay of them indicates that ASIC1a is expressed on the axons. Scale bar, 100 μm. **(B)** co-staining of neurons with anti-GFP (ASIC2a, red) and Neurofilament-H (green) antibodies. Overlay of them indicates that ASIC2a is expressed on the axons. Scale bar, 50 μm.

**FIGURE 6:**
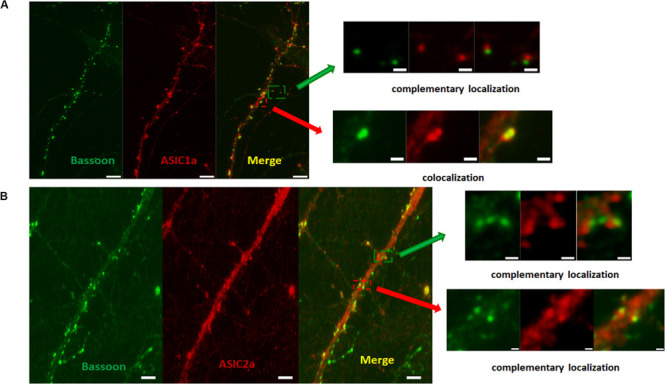
ASICs is expressed on presynaptic membrane in mature hippocampal neurons. **(A)** Presynaptic localization of ASIC1a in DIV14 hippocampal neurons. Right row show magnifications as merged of rectangles in the overview images of the left row. The upper right shows complementary localization of ASIC1a (red) and Bassoon (green). The lower right shows colocalization of ASIC1a (red) and Bassoon (green). Scale bar was 6 μm and 1 μm for the left and right row, respectively. **(B)** Presynaptic localization of ASIC2a in DIV14 hippocampal neurons. Right row show magnifications as merged of rectangles in the overview images of the left row. The upper right shows complementary localization of ASIC2a (red) and Bassoon (green). The lower right shows colocalization of ASIC2a (red) and Bassoon (green). Scale bar was 4 μm and 0.6 μm for the left and right row, respectively.

Our data demonstrated that ASICs are present at axons and functionally expressed in the axonal growth cones of DIV3-DIV6 hippocampal neurons. However, there are paradoxical and unclear reports regarding whether ASICs are present in mature hippocampal neuronal axons. Whether ASICs are expressed in the presynaptic terminal of mature hippocampal synapse also remains unclear. To examine the localization of ASICs at axons and the pre-synapses of mature hippocampal neurons, the ASIC1a subunit and ASIC2a subunit were transduced with AAV-dj-ASIC1a-mcherry and AAV-dj-ASIC2a-eGPF, respectively, in DIV6 hippocampal neurons. Then, DIV14 neurons were immunostained with primary anti-GFP, anti-m-Cherry, anti-Neurofilament-H (axon marker) and anti-Bassoon (presynaptic marker) antibodies. At this stage of *in vitro* neuronal development, hippocampal neurons contain morphologically mature axons and synapses (Grabrucker et al., 2009; Schoen et al., 2015). The transduced ASIC1a and ASIC2a subunits were primarily present in the cell body and dendrites of these mature neurons. Their localization was still visualized at axons, although their expression was greatly reduced (Figure 5). Our results are consistent with previous data that ASIC1a and ASIC2a are located at axons (Alvarez de la Rosa et al., 2003; Zha et al., 2009). Bassoon is a presynaptic scaffolding protein that exhibits presynaptic localization. As shown in Figure 6, both exogenously expressed ASIC1a subunits (red) and ASIC2a subunits (red) were both partly colocalized with Bassoon (green), with ASICs and Bassoon showing three clear patterns of localization (no colocalization, complementary localization, and colocalization). Although the colocation of ASICs and Bassoon accounts for only a small proportion, our data showed that ASICs were expressed in the presynaptic membrane. In summary, our results suggest that ASICs (ASIC1a and ASIC2a) are expressed at mature hippocampal axons and presynaptic terminals.

## Discussion

Our results showed that ASICs (ASIC1a and ASIC2a subunits) are present and functional at the axonal growth cones of young *in vitro* primary cultured hippocampal neurons. The localization of ASICs was elucidated through immunofluorescence colocalization experiments of endogenously and exogenously expressed ASIC subunits. Our electrophysiological recordings and calcium imaging assay results provide functional evidence of ASIC activity at the growth cones of hippocampal neurons. In addition, we also used immunostaining to prove that ASIC1a and ASIC2a subunits are localized throughout the axon and presynaptic active zones.

To the best of our knowledge, this is the first report that ASICs are expressed at the growth cones of immature neurons. Although functional ASIC1a and ASIC2a subunits are located at growth cones, it remains unclear whether the subunits affect growth cone and axon activities. Homomeric ASIC1a channels can permeate Ca^2+^, and intracellular Ca^2+^ signaling is known to be crucial for growth cone activity, including axon extension, turning, growth cone motility and the activation of intracellular signaling pathways. ASIC activation contributes to the activation of the downstream signaling molecule Ca^2+^-sensitive Ca^2+^/calmodulin-dependent protein kinase II (CaMKII) (Gao et al., 2005). CaMKII has been shown to modulate axon growth by affecting F-actin length in growth cone (Xi et al., 2019) and influence growth cone guidance (Wen et al., 2004). In addition, ASICs exhibit functional interaction with N-methyl D-aspartate receptors (NMDARs) to facilitate NMDAR activation (Wemmie et al., 2002; Du et al., 2014; Buta et al., 2015; Liu et al., 2016). NMDARs also mediate Ca^2+^ influx. Numerous signaling molecules implicated in growth cone functions have associations with functionally enhanced NMDARs due to ASIC activation. For example, NMDARs themselves have been implicated in the activity of Cdc42, Rac, and Rho, which have essential roles in developmental mechanisms, including neurite outgrowth, axon guidance, growth cone motility (Dickson, 2001; Wang et al., 2011). Currently, the effect of NMDARs on the function of growth cones is relatively clear, and further research will concentrate on the influence of ASICs on growth cone activity. Whether ASIC channels are distributed in axons and presynaptic terminals is controversial. Regarding the location of ASIC1a receptors, Wemmie et al. showed that ASIC and the axonal marker dephospho-tau-1 were not colocalized, illustrating that ASIC1 is not localized in neuronal axons (Wemmie et al., 2002). In addition, ASIC1 was shown to be colocalized with PSD-95, suggesting that it primarily has postsynaptic localization (Zha et al., 2006). However, Alvarez de la Rosa et al. (2003) observed that in addition to the cell bodies, dendrites and postsynaptic sites, ASIC1 was also located in axons and presynaptic membranes. As to the subcellular localization of ASIC2a, the results of previous studies suggested that a much lower level of ASIC2a was present in neuronal axons (Zha et al., 2009) and no presynaptic expression of ASIC2a was detected in the rat cerebellum (Jovov et al., 2003). There may be many reasons for the above different results, such as differences in antibody quality, cell types, and experimental conditions. We further elucidated the subcellular localization of the ASIC1a and ASIC2a subunits and observed that these subunits are expressed not only in the axons of neurons but also in the presynaptic membrane in cultured mature hippocampal neurons. Although ASIC1a, ASIC2a and Bassoon show mostly complementary distribution patterns, colocalization was observed, though at a low level. However, it is unclear whether their presynaptic localization is functional. From the perspective of functional currents, Gonzalez-Inchauspe et al. (2017) did not detect ASIC-mediated presynaptic currents when presynaptic terminals were patch clamped at −70 mV while applying a pH 5.5 or 6.0 extracellular solution. We believe a possible reason for this finding is that the number of ASICs in presynaptic membranes is too small for their currents to be recorded using whole-cell methods. The presence of these channels can be validated using other more sophisticated methods.

Neurotransmitter release can be influenced by ASICs. The results of previous studies suggested that the probability of glutamate release was increased in ASIC1a^–/–^ mice using microisland cultures of hippocampal neurons (Cho and Askwith, 2008). At the neuromuscular junction, the pharmacological and genetic disruption of ASIC1a activity also enhanced transmitter release (Urbano et al., 2014). However, how ASICs affect neurotransmitter release is currently unclear. One possibility is that postsynaptic ASIC1a could physically interact with a presynaptic counterpart to regulate neurotransmitter release in a retrograde manner (Gonzalez-Inchauspe et al., 2017). Another possibility is that ASICs could directly affect neurotransmitter release, since our results show that ASIC1a and ASIC2a are located in the presynaptic membrane. Further research should focus on studying the function of presynaptic ASICs by obviating the role of postsynaptic ASICs. The release of presynaptic vesicles is associated with Ca^2+^. Although ASIC1a activation can cause Ca^2+^ penetration, previous studies have shown that ASIC1a activation is negatively correlated with presynaptic neurotransmitter release (Cho and Askwith, 2008). These results suggest that presynaptic neurotransmitter release may not be mediated by ASIC1a allowing Ca^2+^ to enter cells in the presynaptic neurons, but other mechanisms could be involved in this process. ASICs could function by modulating other receptors, membrane proteins and ion channels to affect neurotransmitter release. In recent years, protons themselves have been regarded as a kind of neurotransmitter (Du et al., 2014; Gonzalez-Inchauspe et al., 2017) as they are co-released with other neurotransmitter from acidified synaptic vesicles. As autoreceptors, ASICs activation decreases presynaptic neurotransmitter release, perhaps by negative feedback.

Although acid-sensing ion channels are classically described as postsynaptic receptors. However, we observed that in addition to the postsynaptic membrane, a small number of ASIC1a and ASIC2a channels also localize in the presynaptic membrane. During development, ASICs are expressed in axon growth cones. Although the functions of ASICs in axon growth cones and presynaptic terminals are likely different, similar targeting mechanisms could adjust both the early targeting of ASICs to growth cones and the later transport of ASICs to presynaptic terminals. Further studies are required to verify the functional roles of ASICs in axon growth cones and presynaptic terminals and the transition from growth cones to synapses.

## Data Availability Statement

All datasets presented in this study are included in the article/Supplementary Material.

## Ethics Statement

The animal study was reviewed and approved by the Laboratory Animals Center of Beijing Institute of Pharmacology and Toxicology (Beijing, China).

## Author Contributions

XL, CL, JY, SZ, and KW performed the research. XL and RS designed the research. CL and XL analyzed the data. XL wrote the manuscript. RS revised the manuscript. All authors contributed to the article and approved the submitted version.

## Conflict of Interest

The authors declare that the research was conducted in the absence of any commercial or financial relationships that could be construed as a potential conflict of interest.

## Supplementary Material

FIGURE S1 This is a full scan of the entire original gel for Figure 2A.

FIGURE S2 This is a full scan of the entire original gel for Figure 2B.

FIGURE S3 Identification of specificity of ASIC1a and ASIC2a antibodies. **(A)** Detection of ASIC1a protein in cultured hippocampal neurons by double-staining immunofluorescence. Nuclei were immunostained with DAPI (blue). ASIC1a was labeled with FITC (green). **(B)** Detection of ASIC2a protein in cultured hippocampal neurons by double-staining immunofluorescence. Nuclei were immunostained with DAPI (blue). ASIC2a was labeled with FITC (green). ASIC1a and ASIC2a protein do not be detected after pretreatment with the corresponding antigen (Ag) peptide. Scale bars, 20 μm.

## References

[B1] Alvarez de la Rosa D. Krueger S. R. Kolar A. Shao D. Fitzsimonds R. M. Canessa C. M. (2003). Distribution, subcellular localization and ontogeny of ASIC1 in the mammalian central nervous system. *J. Physiol.* 546 77–87. 10.1113/jphysiol.2002.030692 12509480 PMC2342460

[B2] Buta A. Maximyuk O. Kovalskyy D. Sukach V. Vovk M. Ievglevskyi O. (2015). Novel potent orthosteric antagonist of ASIC1a prevents NMDAR-dependent LTP induction. *J. Med. Chem.* 58 4449–4461. 10.1021/jm5017329 25974655

[B3] Chen X. Kalbacher H. Grunder S. (2006). Interaction of acid-sensing ion channel (ASIC) 1 with the tarantula toxin psalmotoxin 1 is state dependent. *J. Gen. Physiol.* 127 267–276. 10.1085/jgp.200509409 16505147 PMC2151504

[B4] Chiang P. H. Chien T. C. Chen C. C. Yanagawa Y. Lien C. C. (2015). ASIC-dependent LTP at multiple glutamatergic synapses in amygdala network is required for fear memory. *Sci. Rep.* 5:10143. 10.1038/srep10143 25988357 PMC4437300

[B5] Cho J. H. Askwith C. C. (2008). Presynaptic release probability is increased in hippocampal neurons from ASIC1 knockout mice. *J. Neurophysiol.* 99 426–441. 10.1152/jn.00940.2007 18094106

[B6] Chu X. P. Xiong Z. G. (2012). Physiological and pathological functions of acid-sensing ion channels in the central nervous system. *Curr. Drug. Targets* 13 263–271. 10.2174/138945012799201685 22204324 PMC3387559

[B7] Dickson B. J. (2001). Rho GTPases in growth cone guidance. *Curr. Opin. Neurobiol.* 11 103–110. 10.1016/s0959-4388(00)00180-x 11179879

[B8] Du J. Reznikov L. R. Price M. P. Zha X. M. Lu Y. Moninger T. O. (2014). Protons are a neurotransmitter that regulates synaptic plasticity in the lateral amygdala. *Proc. Natl. Acad. Sci. U.S.A.* 111 8961–8966. 10.1073/pnas.1407018111 24889629 PMC4066526

[B9] Escoubas P. De Weille J. R. Lecoq A. Diochot S. Waldmann R. Champigny G. (2000). Isolation of a tarantula toxin specific for a class of proton-gated Na+ channels. *J. Biol. Chem.* 275 25116–25121. 10.1074/jbc.M003643200 10829030

[B10] Ettaiche M. Guy N. Hofman P. Lazdunski M. Waldmann R. (2004). Acid-sensing ion channel 2 is important for retinal function and protects against light-induced retinal degeneration. *J. Neurosci.* 24 1005–1012. 10.1523/jneurosci.4698-03.2004 14762118 PMC6793571

[B11] Gao J. Duan B. Wang D. G. Deng X. H. Zhang G. Y. Xu L. (2005). Coupling between NMDA receptor and acid-sensing ion channel contributes to ischemic neuronal death. *Neuron* 48 635–646. 10.1016/j.neuron.2005.10.011 16301179

[B12] Gonzalez-Inchauspe C. Urbano F. J. Di Guilmi M. N. Uchitel O. D. (2017). Acid-sensing ion channels activated by evoked released protons modulate synaptic transmission at the mouse calyx of held synapse. *J. Neurosci.* 37 2589–2599. 10.1523/jneurosci.2566-16.2017 28159907 PMC6596635

[B13] Grabrucker A. Vaida B. Bockmann J. Boeckers T. M. (2009). Synaptogenesis of hippocampal neurons in primary cell culture. *Cell Tissue Res.* 338 333–341. 10.1007/s00441-009-0881-z 19885679

[B14] Grunder S. Pusch M. (2015). Biophysical properties of acid-sensing ion channels (ASICs). *Neuropharmacology* 94 9–18. 10.1016/j.neuropharm.2014.12.016 25585135

[B15] Hruska-Hageman A. M. Wemmie J. A. Price M. P. Welsh M. J. (2002). Interaction of the synaptic protein PICK1 (protein interacting with C kinase 1) with the non-voltage gated sodium channels BNC1 (brain Na+ channel 1) and ASIC (acid-sensing ion channel). *Biochem. J.* 361 443–450. 10.1042/bj3610443 11802773 PMC1222326

[B16] Huang C. Y. Lien C. C. Cheng C. F. Yen T. Y. Chen C. J. Tsaur M. L. (2017). K+ channel Kv3.4 is essential for axon growth by limiting the influx of Ca2+ into growth cones. *J. Neurosci.* 37 4433–4449. 10.1523/jneurosci.1076-16.2017 28320840 PMC6596659

[B17] Jiang N. Wu J. Leng T. Yang T. Zhou Y. Jiang Q. (2017). Region specific contribution of ASIC2 to acidosis-and ischemia-induced neuronal injury. *J. Cereb. Blood Flow Metab.* 37 528–540. 10.1177/0271678x16630558 26861816 PMC5381448

[B18] Jovov B. Tousson A. McMahon L. L. Benos D. J. (2003). Immunolocalization of the acid-sensing ion channel 2a in the rat cerebellum. *Histochem. Cell. Biol.* 119 437–446. 10.1007/s00418-003-0525-4 12768285

[B19] Kellenberger S. Schild L. (2015). International union of basic and clinical pharmacology. XCI. structure, function, and pharmacology of acid-sensing ion channels and the epithelial Na+ channel. *Pharmacol. Rev.* 67 1–35. 10.1124/pr.114.009225 25287517

[B20] Kreple C. J. Lu Y. Taugher R. J. Schwager-Gutman A. L. Du J. Stump M. (2014). Acid-sensing ion channels contribute to synaptic transmission and inhibit cocaine-evoked plasticity. *Nat. Neurosci.* 17 1083–1091. 10.1038/nn.3750 24952644 PMC4115047

[B21] Li W. G. Liu M. G. Deng S. Liu Y. M. Shang L. Ding J. (2016). ASIC1a regulates insular long-term depression and is required for the extinction of conditioned taste aversion. *Nat. Commun.* 7:13770. 10.1038/ncomms13770 27924869 PMC5150990

[B22] Lingueglia E. de Weille J. R. Bassilana F. Heurteaux C. Sakai H. Waldmann R. (1997). A modulatory subunit of acid sensing ion channels in brain and dorsal root ganglion cells. *J. Biol. Chem.* 272 29778–29783. 10.1074/jbc.272.47.29778 9368048

[B23] Liu M. G. Li H. S. Li W. G. Wu Y. J. Deng S. N. Huang C. (2016). Acid-sensing ion channel 1a contributes to hippocampal LTP inducibility through multiple mechanisms. *Sci. Rep.* 6:23350. 10.1038/srep23350 26996240 PMC4800407

[B24] Mango D. Nistico R. (2019). Acid-sensing ion channel 1a is involved in N-Methyl D-aspartate receptor-dependent long-term depression in the hippocampus. *Front. Pharmacol.* 10:555. 10.3389/fphar.2019.00555 31178731 PMC6537656

[B25] Price M. P. Gong H. Parsons M. G. Kundert J. R. Reznikov L. R. Bernardinelli L. (2014). Localization and behaviors in null mice suggest that ASIC1 and ASIC2 modulate responses to aversive stimuli. *Genes. Brain Behav.* 13 179–194. 10.1111/gbb.12108 24256442 PMC3998777

[B26] Ruscher K. Freyer D. Karsch M. Isaev N. Megow D. Sawitzki B. (2002). Erythropoietin is a paracrine mediator of ischemic tolerance in the brain: evidence from an in vitro model. *J. Neurosci.* 22 10291–10301. 10.1523/jneurosci.22-23-10291.2002 12451129 PMC6758760

[B27] Schoen M. Reichel J. M. Demestre M. Putz S. Deshpande D. Proepper C. (2015). Super-resolution microscopy reveals presynaptic localization of the ALS/FTD related protein FUS in hippocampal neurons. *Front. Cell Neurosci.* 9:496. 10.3389/fncel.2015.00496 26834559 PMC4709451

[B28] Urbano F. J. Lino N. G. Gonzalez-Inchauspe C. M. Gonzalez L. E. Colettis N. Vattino L. G. (2014). Acid-sensing ion channels 1a (ASIC1a) inhibit neuromuscular transmission in female mice. *Am. J. Physiol. Cell. Physiol.* 306 C396–C406. 10.1152/ajpcell.00301.2013 24336653 PMC3919981

[B29] Waldmann R. Champigny G. Bassilana F. Heurteaux C. Lazdunski M. (1997). A proton-gated cation channel involved in acid-sensing. *Nature* 386 173–177. 10.1038/386173a0 9062189

[B30] Wang P. Y. Petralia R. S. Wang Y. X. Wenthold R. J. Brenowitz S. D. (2011). Functional NMDA receptors at axonal growth cones of young hippocampal neurons. *J. Neurosci.* 31 9289–9297. 10.1523/jneurosci.5639-10.2011 21697378 PMC3124703

[B31] Wemmie J. A. Askwith C. C. Lamani E. Cassell M. D. Freeman J. H. Jr. (2003). Acid-sensing ion channel 1 is localized in brain regions with high synaptic density and contributes to fear conditioning. *J. Neurosci.* 23 5496–5502. 10.1523/jneurosci.23-13-05496.2003 12843249 PMC6741257

[B32] Wemmie J. A. Chen J. Askwith C. C. Hruska-Hageman A. M. Price M. P. Nolan B. C. (2002). The acid-activated ion channel ASIC contributes to synaptic plasticity, learning, and memory. *Neuron* 34 463–477. 10.1016/s0896-6273(02)00661-x 11988176

[B33] Wemmie J. A. Coryell M. W. Askwith C. C. Lamani E. Leonard A. S. Sigmund C. D. (2004). Overexpression of acid-sensing ion channel 1a in transgenic mice increases acquired fear-related behavior. *Proc. Natl. Acad. Sci. U.S.A.* 101 3621–3626. 10.1073/pnas.0308753101 14988500 PMC373512

[B34] Wemmie J. A. Taugher R. J. Kreple C. J. (2013). Acid-sensing ion channels in pain and disease. *Nat. Rev. Neurosci.* 14 461–471. 10.1038/nrn3529 23783197 PMC4307015

[B35] Wen Z. Guirland C. Ming G. L. Zheng J. Q. (2004). A CaMKII/calcineurin switch controls the direction of Ca2+-dependent growth cone guidance. *Neuron* 43 835–846. 10.1016/j.neuron.2004.08.037 15363394

[B36] Xi F. Xu R. J. Xu J. H. Ma J. J. Wang W. H. Wang F. (2019). Calcium/calmodulin-dependent protein kinase II regulates mammalian axon growth by affecting F-actin length in growth cone. *J. Cell. Physiol.* 234 23053–23065. 10.1002/jcp.28867 31134625

[B37] Xiong Z. G. Chu X. P. Simon R. P. (2006). Ca2+ -permeable acid-sensing ion channels and ischemic brain injury. *J. Membr. Biol.* 209 59–68. 10.1007/s00232-005-0840-x 16685601

[B38] Xiong Z. G. Zhu X. M. Chu X. P. Minami M. Hey J. Wei W. L. (2004). Neuroprotection in ischemia: blocking calcium-permeable acid-sensing ion channels. *Cell* 118 687–698. 10.1016/j.cell.2004.08.026 15369669

[B39] Yu Z. Wu Y. J. Wang Y. Z. Liu D. S. Song X. L. Jiang Q. (2018). The acid-sensing ion channel ASIC1a mediates striatal synapse remodeling and procedural motor learning. *Sci. Signal.* 11:eaar4481. 10.1126/scisignal.aar4481 30087178 PMC6324561

[B40] Zha X. M. Costa V. Harding A. M. Reznikov L. Benson C. J. Welsh M. J. (2009). ASIC2 subunits target acid-sensing ion channels to the synapse via an association with PSD-95. *J. Neurosci.* 29 8438–8446. 10.1523/jneurosci.1284-09.2009 19571134 PMC2734339

[B41] Zha X. M. Wemmie J. A. Green S. H. Welsh M. J. (2006). Acid-sensing ion channel 1a is a postsynaptic proton receptor that affects the density of dendritic spines. *Proc. Natl. Acad. Sci. U.S.A.* 103 16556–16561. 10.1073/pnas.0608018103 17060608 PMC1621052

[B42] Zhang H. Gao G. Zhang Y. Sun Y. Li H. Dong S. (2017). Glucose deficiency elevates acid-sensing ion channel 2a expression and increases seizure susceptibility in temporal lobe epilepsy. *Sci. Rep.* 7:5870. 10.1038/s41598-017-05038-0 28725010 PMC5517604

[B43] Ziemann A. E. Allen J. E. Dahdaleh N. S. Drebot I. I. Coryell M. W. Wunsch A. M. (2009). The amygdala is a chemosensor that detects carbon dioxide and acidosis to elicit fear behavior. *Cell* 139 1012–1021. 10.1016/j.cell.2009.10.029 19945383 PMC2808123

[B44] Ziemann A. E. Schnizler M. K. Albert G. W. Severson M. A. Howard M. A. Welsh M. J. (2008). Seizure termination by acidosis depends on ASIC1A. *Nat. Neurosci.* 11 816–822. 10.1038/nn.2132 18536711 PMC2553357

