# Supplementary figures and images for: Distribution of Acid Sensing Ion Channels in Axonal Growth Cones and Presynaptic Membrane of Cultured Hippocampal Neurons
Source: Front Cell Neurosci. 2020 Jul 7;14:205. doi: 10.3389/fncel.2020.00205 (PMC7358772; doi:10.3389/fncel.2020.00205)

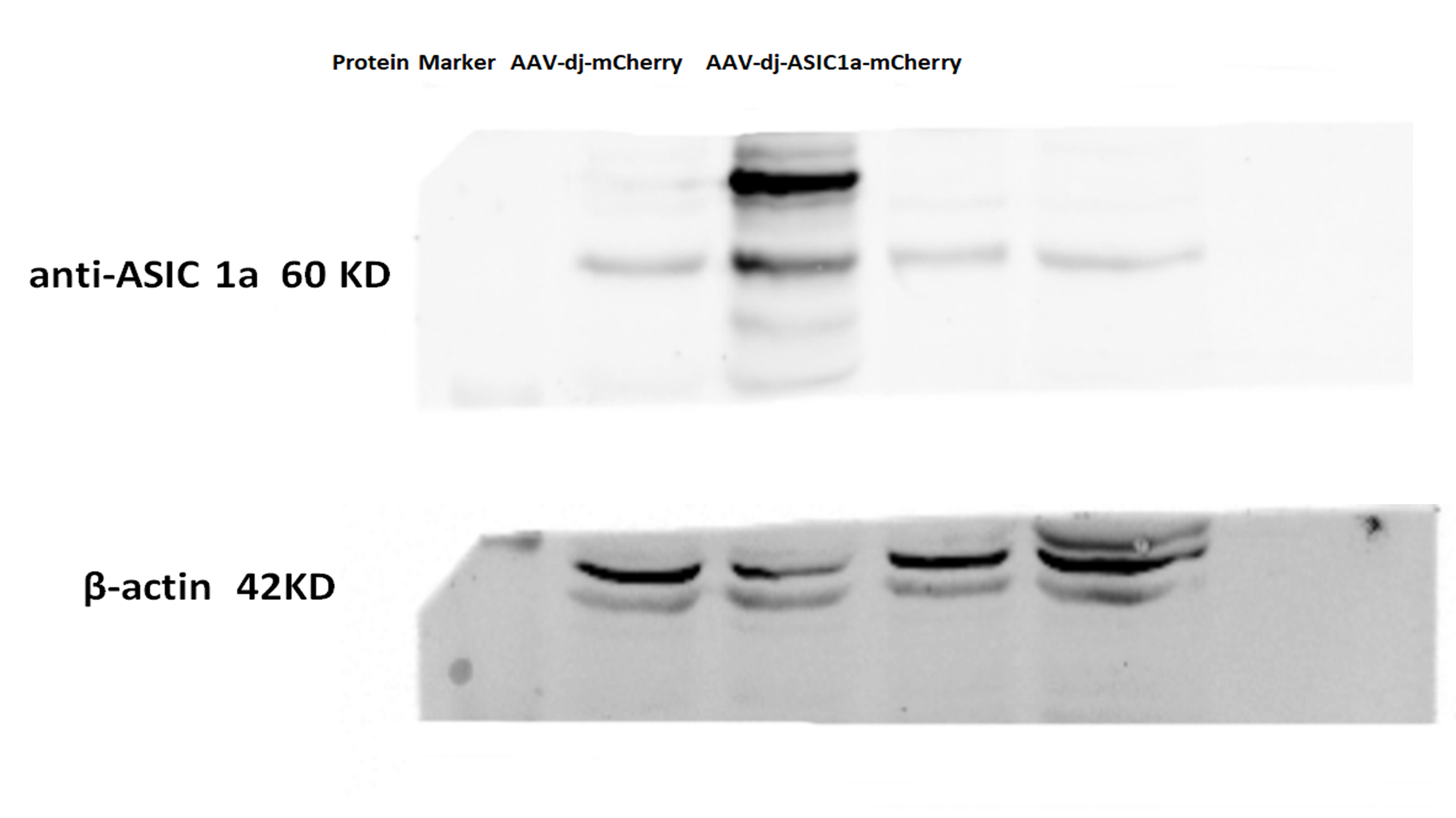

Supplement: FIGURE S1 — This is a full scan of the entire original gel for Figure 2A. [file Image_1.TIF]
